# Shedding new light on circadian clocks

**DOI:** 10.7554/eLife.00659

**Published:** 2013-04-09

**Authors:** Maud Demarque, Ueli Schibler

**Affiliations:** 1 **Maud Demarque** is at the Department of Molecular Biology, University of Geneva, Geneva, Switzerlandmaud.demarque@unige.ch; 2 **Ueli Schibler** is at the Department of Molecular Biology, University of Geneva, Geneva, Switzerlandueli.schibler@unige.ch

**Keywords:** Clock gene, USF1, genetic modifier, circadian rhythms, mouse genetics, ChIP-Seq, Mouse

## Abstract

Using a clever combination of genetic and biochemical tools researchers have shown that a transcription factor called USF1 has a central role in determining how mutations of the *Clock* gene manifest themselves in the behaviour of different mouse strains.

**Related research article** Shimomura K, Kumar V, Koike N, Kim T-K, Chong J, Buhr ED, Whiteley AR, Low SS, Omura C, Fenner D, Owens JR, Richards M, Yoo S-H, Hong H-K, Vitaterna MH, Bass J, Pletcher MT, Wiltshire T, Hogenesch J, Lowrey PL, Takahashi JS. 2013. *Usf1*, a suppressor of the circadian *Clock* mutant, reveals the nature of the DNA-binding of the CLOCK:BMAL1 complex in mice. *eLife* **2**:e00426. doi: 10.7554/eLife.00426

**Image** Heat maps comparing how three transcription factors bind to DNA in wild-type and mutant mice

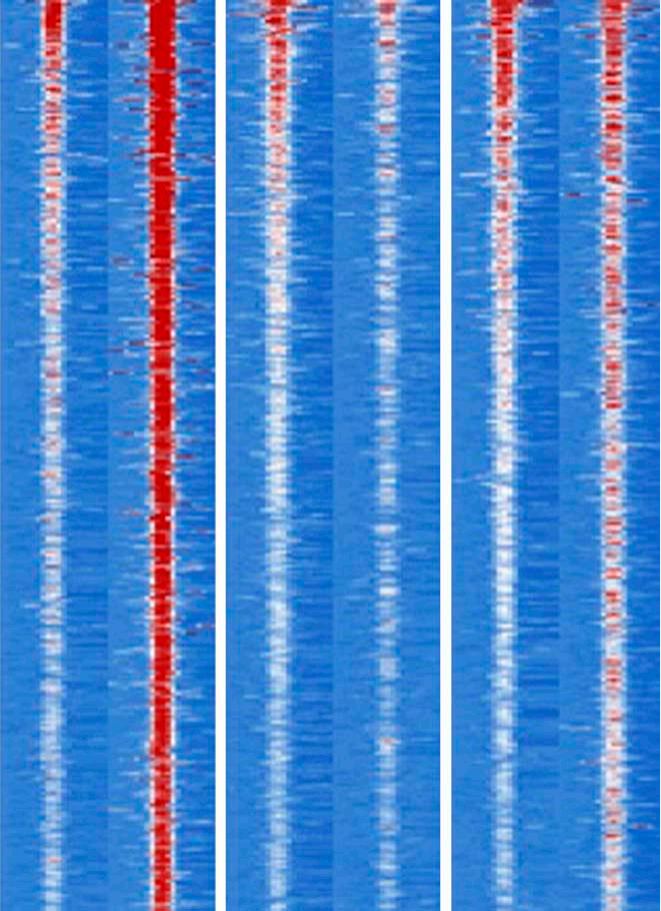

Circadian clocks adapt the physiology of many different species to recurring changes in their environments—notably, to 24-hour cycles of daylight and darkness—in a proactive manner. In mammals, the circadian timing system is composed of a master pacemaker in the suprachiasmatic nucleus, which is located in the brain, and subsidiary clocks in virtually all body cells. However, these circadian clocks can measure time only approximately—the Latin words circa diem mean ‘about a day'—so they must be synchronized daily to ensure that they remain in time with the 24-hour day.

The process of synchronization begins with electrical signals from the retina causing an influx of calcium ions into postsynaptic neurons in the suprachiasmatic nucleus. This influx activates various protein kinases that increase the activity of a number of immediate early transcription factors which, in turn, enhance the expression of genes ensuring that the master pacemaker remains synchronized. The suprachiasmatic nucleus then synchronizes the subsidiary clocks via a large variety of signalling pathways that depend on feeding cycles, hormones, body temperature rhythms and neuronal cues (Dibner et al., 2010).

The molecular circuitry responsible for maintaining circadian rhythms is composed of two interlocked feedback loops. The major loop relies on two transcription factors, CLOCK and BMAL1, forming a complex that stimulates the expression of target genes by binding to regions of DNA called Enhancer-boxes (E-boxes) that are associated with the target genes (Figure 1). Four of these target genes—*Per1*, *Per2*, *Cry1* and *Cry2*—produce proteins that counteract the effects of the CLOCK:BMAL1 complex and, as a consequence, establish a negative feedback loop that actually suppresses their own expression (Lowrey and Takahashi, 2000; Reppert and Weaver, 2001). In the other loop, CLOCK and BMAL1 control the transcription of the genes that code for various nuclear receptors that govern the cyclic transcription of the *Bmal1* and *Clock* genes (Preitner et al., 2002; Ueda et al., 2002; Bugge et al., 2012; Cho et al., 2012). The circadian system also depends on a large number of post-translational processes. The overall effect of these two feedback loops, plus the various post-translational processes, is to determine the intrinsic period of the circadian cycle, which is subsequently synchronized to a period of 24 hours as described above.

**Figure 1.**
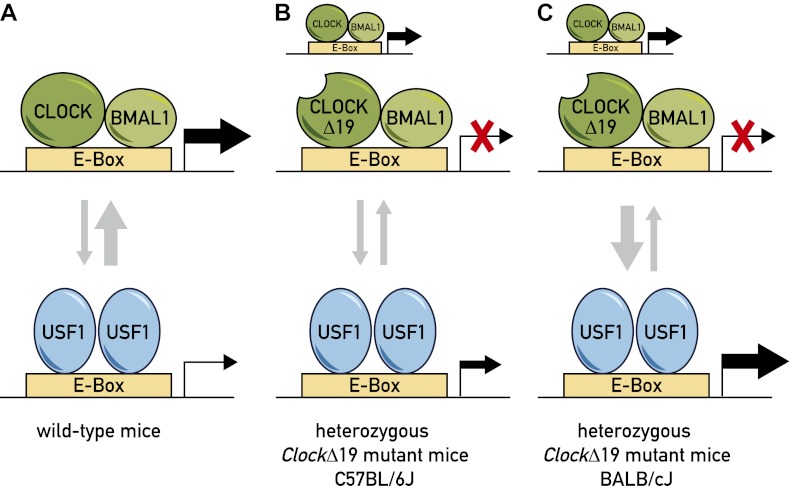
Model for the role of two transcription factors—CLOCK and USF1—in the circadian clock. (**A**) In wild-type mice, the CLOCK:BMAL1 complex binds to E-box sites on the genome with a much higher affinity than USF1, so they occupy the majority of E-box sites (as denoted by the relative thicknesses of the vertical arrows). This means that CLOCK:BMAL1 complexes also have a much larger role in the transcription of the gene associated with the E-box (as denoted by the relative thicknesses of the horizontal arrows). (**B**) In some *ClockΔ19* mutant mice (such as heterozygous C57BL/6J mice), both the CLOCK:BMAL1 and CLOCKΔ19:BMAL1 complexes bind to E-box sites, but the latter is not able to drive transcription (denoted by the red X). In these mice the total level of transcription driven by the CLOCK:BMAL1 complex and USF1 is not sufficient to drive normal circadian gene expression, which results in the period of the circadian cycle becoming longer. (**C**) Other *ClockΔ19* mutant mice (such as heterozygous BALB/cJ mice) produce higher levels of USF1 than C57BL/6J mice (denoted by the thick arrow pointing down), and the total level of transcription driven by the CLOCK:BMAL1 complex and USF1 is high enough to drive normal circadian gene expression.

In the 1990s, researchers led by Joseph Takahashi—then at Northwestern University, now at the University of Texas Southwestern (UTSW) Medical Center—identified a semi-dominant mutation in the *Clock* gene, *ClockΔ19*, that lengthened the circadian period in mice of the C57BL/6J strain, and caused most homozygous mutant mice to become arrhythmic when exposed to constant darkness over an extended time period (Antoch et al., 1997; King et al., 1997). Although the CLOCKΔ19 mutant protein can still form a complex with BMAL1 that is capable of binding to DNA (albeit with reduced affinity), it is not able to activate transcription. Takahashi and colleagues also noted that the penetrance of the clock mutant phenotype (that is, the fraction of mice in which these traits were evident) was much higher in some strains than in others. In order to identify the genes that might be responsible for this difference between strains, they performed a complex trait analysis on nearly 200 hybrid mice and identified 14 loci associated with possible *ClockΔ19* modulator genes (Shimomura et al., 2001).

Now, in *eLife*, Takahashi and co-workers—including Kazuhiro Shimomura of Northwestern University as first author—report that they have extended this work by identifying a transcription factor that suppresses the *ClockΔ19* mutation in one strain of these mice (a strain called BALB/cJ). To this end they used a combination of two genetic techniques (quantitative trait locus and haplotype block mapping) to narrow down the region of the genome that is responsible for the suppression of this mutation: this region is a DNA segment encompassing about 900 kilo base pairs and 22 protein encoding genes. Seven of these genes were expressed in the same tissues as *Clock*. However, only one of them, *Usf1* (which codes for a transcription factor called upstream transcription factor 1), was more active in mice in which the *ClockΔ19* mutation was suppressed. Moreover, a moderate overexpression of USF1 suppressed the *ClockΔ19* mutant phenotype in C57BL/6J mice. This provides compelling evidence for the scenario proposed by the authors, namely that the increased accumulation of USF1 in BALB/cJ mice accounts for the suppression of the *ClockΔ19* mutant phenotype in this mouse strain.

The region responsible for increased transcription of the *Usf1* gene in BALB/cJ mice has been mapped to a DNA fragment containing about 1000 base pairs. Importantly, the analysis of protein-DNA complexes by biochemical assays and chromatin-immunoprecipitation experiments demonstrated that USF1 and CLOCK:BMAL1 can indeed bind to the same E-box motifs.

The intrinsic period of the circadian cycle can be determined by keeping the mice under conditions of constant darkness. Such experiments reveal that *Usf1* knockout mice and wild-type mice have similar intrinsic periods. Thus, USF1 does not contribute significantly to determining the period of the circadian cycle in wild-type mice. However, compared to wild-type mice, the mutant mice are less active overall. Moreover, their levels of activity vary much less over the circadian cycle. This suggests that USF1 contributes to the robustness of circadian behaviour.

Based on an extensive body of genetic and biochemical work, Takahashi, Shimomura and co-workers have shown that USF1 is involved in the fine-tuning of the circadian system. Moreover, as USF1 does not appear to require BMAL1 to recognize E-box DNA elements, it is likely that the CLOCK:BMAL1 complex and USF1 cannot bind their cognate DNA elements at the same time, at least in target genes that depend on a single E-box (Figure 1). Shimomura et al. thus suggest that USF1 may facilitate the binding of CLOCK:BMAL1 complexes by maintaining an open chromatin configuration around the relevant E-box.

In addition to the impact that this work will have on research into circadian rhythms, it also addresses an issue—penetrance—that is of utmost importance for complex genetics. A large fraction of illnesses, including cardiovascular dysfunction, metabolic syndrome, cancer and degenerative brain diseases, are caused by complex interactions between multiple alleles and modifier genes. However, only a few of the genes that influence the penetrance of a mutant allele have been cloned and dissected at the molecular level. The work of Shimomura et al. provides a convincing example for how genetic penetrance can be modulated in different genetic backgrounds. Indeed, it goes far beyond the analysis of circadian clocks and is relevant for a large community of life scientists interested in genetic networks.

## References

[bib1] Antoch MP Song EJ Chang AM Vitaterna MH Zhao Y Wilsbacher LD 1997 Functional identification of the mouse circadian clock gene by transgenic BAC rescue. Cell 89:655–67 doi: 10.1016/S0092-8674(00)80246-9 9160756 PMC3764491

[bib2] Bugge A Feng D Everett LJ Briggs ER Mullican SE Wang F 2012 Rev-erbα and Rev-erbβ coordinately protect the circadian clock and normal metabolic function. Genes Dev 26:657–67 doi: 10.1101/gad.186858.112 22474260 PMC3323877

[bib3] Cho H Zhao X Hatori M Yu RT Barish GD Lam MT 2012 Regulation of circadian behaviour and metabolism by REV-ERB-α and REV-ERB-β. Nature 485:123–7 doi: 10.1038/nature11048 22460952 PMC3367514

[bib4] Dibner C Schibler U Albrecht U 2010 The mammalian circadian timing system: organization and coordination of central and peripheral clocks. Annu Rev Physiol 72:517–49 doi: 10.1146/annurev-physiol-021909-135821 20148687

[bib5] King DP Zhao Y Sangoram AM Wilsbacher LD Tanaka M Antoch MP 1997 Positional cloning of the mouse circadian clock gene. Cell 89:641–53 doi: 10.1016/S0092-8674(00)80245-7 9160755 PMC3815553

[bib6] Lowrey PL Takahashi JS 2000 Genetics of the mammalian circadian system: photic entrainment, circadian pacemaker mechanisms, and post-translational regulation. Annu Rev of Genet 34:533–62 doi: 10.1146/annurev.genet.34.1.533 11092838

[bib7] Preitner N Damiola F Lopez-Molina L Zakany J Duboule D Albrecht U 2002 The orphan nuclear receptor REV-ERBα controls circadian transcription within the positive limb of the mammalian circadian oscillator. Cell 110:251–60 doi: 10.1016/S0092-8674(02)00825-5 12150932

[bib8] Reppert SM Weaver DR 2001 Molecular analysis of mammalian circadian rhythms. Annu Rev Physiol 63:647–76 doi: 10.1146/annurev.physiol.63.1.647 11181971

[bib9] Shimomura K Low-Zeddies SS King DP Steeves TD Whiteley A Kushla J 2001 Genome-wide epistatic interaction analysis reveals complex genetic determinants of circadian behavior in mice. Genome Res 11:959–80 doi: 10.1101/gr.171601 11381025

[bib10] Shimomura K Kumar V Koike N Kim T-K Chong J Buhr ED 2013 *Usf1*, a suppressor of the circadian *Clock* mutant, reveals the nature of the DNA-binding of the CLOCK:BMAL1 complex. eLife 2:e00426 doi: 10.7554/elife.00426 PMC3622178 23580255

[bib11] Ueda HR Chen W Adachi A Wakamatsu H Hayashi S Takasugi T 2002 A transcription factor response element for gene expression during circadian night. Nature 418:534–9 doi: 10.1038/nature00906 12152080

